# Intra-Arterial MR Perfusion Imaging of Meningiomas: Comparison to Digital Subtraction Angiography and Intravenous MR Perfusion Imaging

**DOI:** 10.1371/journal.pone.0163554

**Published:** 2016-11-01

**Authors:** Mark A. Lum, Alastair J. Martin, Matthew D. Alexander, David B. McCoy, Daniel L. Cooke, Prasheel Lillaney, Parham Moftakhar, Matthew R. Amans, Fabio Settecase, Andrew Nicholson, Christopher F. Dowd, Van V. Halbach, Randall T. Higashida, Michael W. McDermott, David Saloner, Steven W. Hetts

**Affiliations:** 1 School of Medicine, University of California San Francisco, San Francisco, California, United States of America; 2 Department of Radiology and Biomedical Imaging, University of California San Francisco, San Francisco, California, United States of America; 3 Department of Neurological Surgery, University of California San Francisco, San Francisco, California, United States of America; 4 Department of Neurology, University of California San Francisco, San Francisco, California, United States of America; 5 Department of Anesthesia and Perioperative Care, University of California San Francisco, San Francisco, California, United States of America; Ohio State University Wexner Medical Center, UNITED STATES

## Abstract

**Background and Purpose:**

To evaluate the ability of IA MR perfusion to characterize meningioma blood supply.

**Methods:**

Studies were performed in a suite comprised of an x-ray angiography unit and 1.5T MR scanner that permitted intraprocedural patient movement between the imaging modalities. Patients underwent intra-arterial (IA) and intravenous (IV) T2* dynamic susceptibility MR perfusion immediately prior to meningioma embolization. Regional tumor arterial supply was characterized by digital subtraction angiography and classified as external carotid artery (ECA) dural, internal carotid artery (ICA) dural, or pial. MR perfusion data regions of interest (ROIs) were analyzed in regions with different vascular supply to extract peak height, full-width at half-maximum (FWHM), relative cerebral blood flow (rCBF), relative cerebral blood volume (rCBV), and mean transit time (MTT). Linear mixed modeling was used to identify perfusion curve parameter differences for each ROI for IA and IV MR imaging techniques. IA vs. IV perfusion parameters were also directly compared for each ROI using linear mixed modeling.

**Results:**

18 ROIs were analyzed in 12 patients. Arterial supply was identified as ECA dural (n = 11), ICA dural (n = 4), or pial (n = 3). FWHM, rCBV, and rCBF showed statistically significant differences between ROIs for IA MR perfusion. Peak Height and FWHM showed statistically significant differences between ROIs for IV MR perfusion. RCBV and MTT were significantly lower for IA perfusion in the Dural ECA compared to IV perfusion. Relative CBF in IA MR was found to be significantly higher in the Dural ICA region and MTT significantly lower compared to IV perfusion.

## Introduction

Meningiomas are highly vascular neoplasms derived from the meninges of the central nervous system. While most meningiomas are benign, their location or size may cause neurologic symptoms requiring resection[1]. Preoperative embolization of meningiomas can safely reduce blood loss and the need for intraoperative transfusion during resection[2–4]. Not all meningiomas are amenable to embolization, however, and nontarget embolization can cause neurologic deficits as a result of ischemia[5]. Whereas dural arteries supplying meningiomas are often amenable to embolization, pial arteries supplying meningiomas are generally unsafe to embolize, as they also supply brain tissue. Although most branches of the ICA that supply meningiomas are pial, some are purely dural in supply, including distal branches of the meningohypophyseal trunk, inferolateral trunk, anterior deep temporal artery, or ophthalmic artery, and are potentially safe and helpful to embolize[6]. Conversely, although most ECA branches supplying meningiomas are dural, some of these supply eloquent structures like the facial nerve that preclude safe embolization[7]. Differentiating not only ICA versus ECA blood supply but also pial versus dural blood supply is essential in determining the feasibility of embolization. This currently requires preoperative evaluation with selective digital subtraction angiography (DSA).

Perfusion MRI is an emerging imaging modality that can provide quantitative information based on MR properties. MR perfusion has been used in a variety of clinical situations including evaluation of stroke[8], grading of gliomas[9, 10], and differentiation of benign and malignant meningiomas[11]. Dynamic susceptibility contrast (DSC) MR perfusion involves injection of gadolinium contrast and can provide quantitative information such as MTT, CBV, and CBF. In addition, ECA and ICA supplies can be differentiated based on bolus transit properties, which are affected by differences in blood brain barrier permeability[12]. In contrast to standard planar DSA, DSC MR perfusion can directly visualize brain and tumor tissue and allows quantitative image analysis. If MR perfusion can accurately demonstrate non-embolizable tumors based on the source of the blood supply, it may prevent nontarget embolization and more sensitively detect residual vascularized tumor following embolization than DSA can.

In contradistinction to the large literature on intravenous (IV) MR perfusion, few studies have examined the efficacy of IA MR perfusion in the assessment of meningioma vasculature since it requires the use of MRI-safe catheters. The aim of the study is to compare the accuracy of IA and IV MR perfusion to DSA in evaluation of meningioma blood supply. We hypothesize that IV and IA MR perfusion will identify pial blood supply as demonstrated by more rapid signal recovery on perfusion curves.

## Materials and Methods

### Subjects

This HIPAA-compliant study was approved by the Committee on Human Research of the Human Research Protection Program at the University of California San Francisco (IRB Approval Number H5570-23757). All participants provided written informed consent, and all consent forms were stored in a secure location. All procedures performed following obtaining consent were approved by the IRB. Patients who underwent surgical resection of their meningioma within 2 weeks of preoperative embolization between September 2006 and July 2011 were studied. Informed consent was obtained, and participants were screened for contraindications to the procedures.

### XMR Suite

All patients underwent baseline MRI including IV MR perfusion at least 1 day prior to their endovascular procedure. Imaging was performed in a combined x-ray and MRI suite comprised of an x-ray angiography unit (Philips Integris V5000, Cleveland, OH) and 1.5T MR scanner (Philips Achieva). The combined XMR suite permitted rapid intraprocedural patient movement between imaging modalities.

### Intravenous MR Perfusion

Perfusion weighted DSC imaging was performed with a single-shot echo-planar T_2_*-weighted acquisition (TR/TE/flip angle, 2000 ms/50 ms/90°; epi factor, 89; FOV, 24 cm; matrix, 128x89; slices, 12–5 mm; number of dynamics, 60; axial plane; acquisition time, 2 min 6 s). The acquisition was designed to acquire >20s (10 dynamics) baseline data prior to contrast arrival and to continue for approximately 1 minute after contrast arrival. Gadolinium based contrast (gadodiamide, GE Healthcare) was injected intravenously either through an antecubital or hand vein. Contrast was injected at either 4 cc/s (antecubital) or 3 cc/s (hand) to a dose of 0.2 mmol/kg (typically ~20ml). The contrast was followed by a 15 ml saline push at the same injection rate.

### Angiographic Procedure

Vascular access was achieved with a transfemoral approach by the Seldinger technique. All patients received 2000 units of intravenous heparin to mitigate risk of clot formation. A specific catheter was used in all studies (5F Cook Beacon Tip Torcon Advantage Catheter, Bloomington, IN) that has previously been tested for safety in the MR environment and under imaging conditions consistent with MR perfusion imaging[13]. Vascular anatomy was determined by DSA in combination with selective injection of iodinated contrast into the vertebral arteries and external and internal carotid arteries. This was performed bilaterally for all patients irrespective of meningioma location. Super-selective microcatheter angiograms of vessels such as the middle meningeal artery were performed as warranted by these initial findings. An interventional neuroradiologist (S.W.H., 9 years experience) qualitatively assessed the fractional contributions of all vessels supplying the tumor. This assessment was done without knowledge of MR findings.

### Intra-arterial MR Perfusion

In order to preclude the need for patient movement while catheterized, a flexible two-element surface coil array consisting of two 20 cm circular loops was applied for all IA imaging. These coils were placed laterally against the patients’ heads and secured in place with tape. IA contrast injections were performed with contrast diluted in physiologic saline to 50mM for all studies (1:10 dilution). IA injections were performed by a power injector through a catheter pre-loaded with the appropriate solution, with injection rates of either 1.0 ml/s (ECA and vertebral artery) or 3.0 ml/s (common carotid artery, CCA). Injection durations of 5 seconds were maintained to provide a bolus width comparable to IV injections and in balance with the temporal resolution of the whole brain perfusion acquisition (2 seconds). These injection rates are also comparable to those utilized for x-ray angiographic purposes. IA injections commenced 20 seconds after the start of the first dynamic scan since there is very little delay between injection and arrival of the contrast agent in distal tissue. No saline push was performed, which was different from the IV perfusion methodology, as this is not standard angiographic practice. Patients receiving therapy via the ECA had perfusion scans performed in both the ECA and subsequently in the CCA. This was accomplished by initially placing the catheter in the ECA under x-ray guidance and then retracting the catheter approximately 5 cm to enter the CCA when the patient was in the MR scanner. Perfusion sequences were performed with the same parameters as IV perfusion sequences.

### Perfusion Analysis

**Fig 1:**
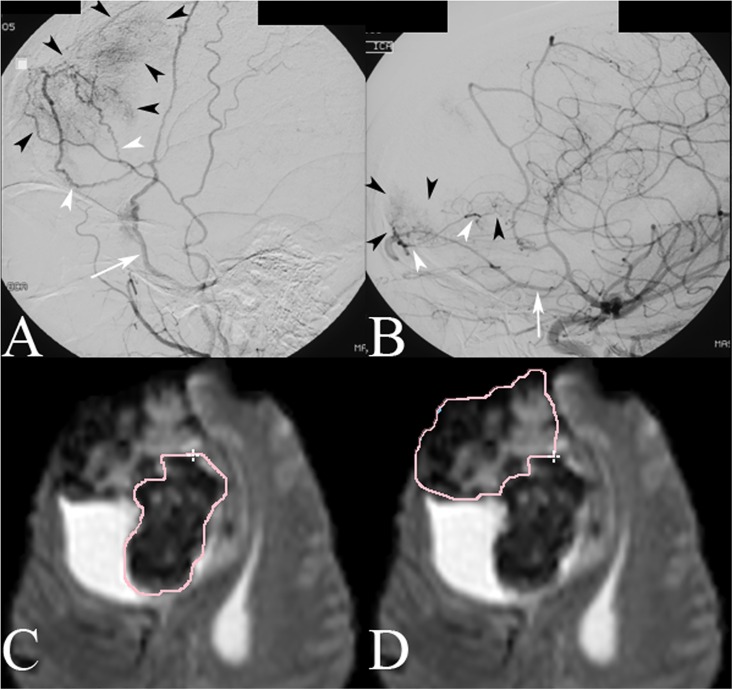
DSA in the lateral projection during injection of the right ECA (A) demonstrates vascular blush (black arrowheads) from a right frontal meningioma supplied by anterior division branches (white arrowheads) of the right middle meningeal artery (white arrow). DSA during injection of the right ICA (B) demonstrates vascular blush (black arrowheads) to the anteroinferior component of the tumor from pial branches (white arrowheads) of the frontopolar branch (white arrow) of the right anterior cerebral artery. Perfusion ROIs for dural (C) and pial (C) contributions to the tumor based on DSA findings.

Perfusion analysis was performed with the Philips Neuro Perfusion package (Philips Extended MR Workspace R2.6.3.3). Based on DSA data, regions-of-interest (ROIs) were uniformly drawn on pre-operative CCA perfusion images representing ECA dural, ICA dural, and pial supplies. ROIs were hand drawn on equivalent slices for IV and IA studies. From these ROIs, perfusion curves were generated, and the following parameters extracted: peak height (expressed as quotient of peak height divided by maximum height), full-width at half-maximum (FWHM), rCBV, rCBF and MTT. ROIs were drawn in ImageJ (National Institutes of Health, Bethesda, Maryland). For lesions deemed to have fractional contribution, separate ROIs were drawn for dural and pial supply by first drawing an ROI for dural supply and then drawing an ROI for pial supply consisting of the remainder of the lesion not included in the dural supply ROI (Fig 1).

### Statistical Analysis

A Shapiro-Wilk’s test (p>.05) was used to determine if each of the perfusion curve parameter variables were normally distributed. Because several patients were repeatedly measured, and because of the low sample size in both the pial ROI group (n = 3) and dural ICA group (n = 4), linear mixed effects models were used with fixed effects for each perfusion parameter by ROI group and random effects for individual patients in order to detect perfusion differences between ROIs for IA and IV imaging. Differences for each perfusion parameter between IV and IA perfusion were also measured using mixed effects modeling with fixed effects for perfusion parameters by imaging type (IV or IA) and random effects for each patient. Marginal predictions were calculated from mixed modeling for variables indicating significant (alpha = .05) test results. P-values and standard errors for differences between ROIs for each imaging type were calculated using linear combinations of regression coefficients for each perfusion parameter.

## Results

**Fig 2:**
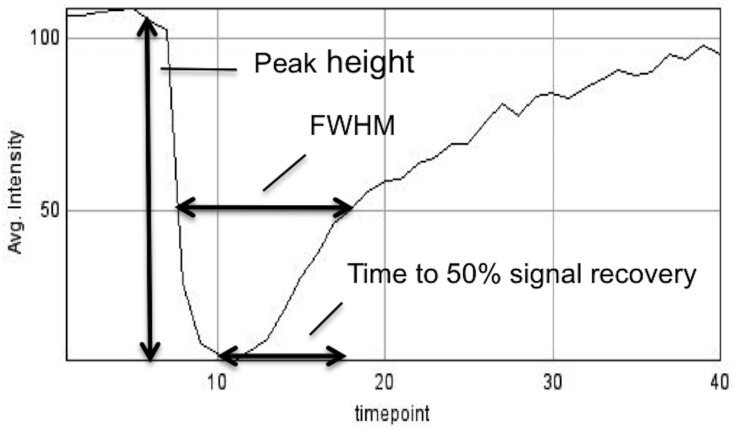
Perfusion curve parameters for sample curve derived from a dural ROI.

**Fig 3:**
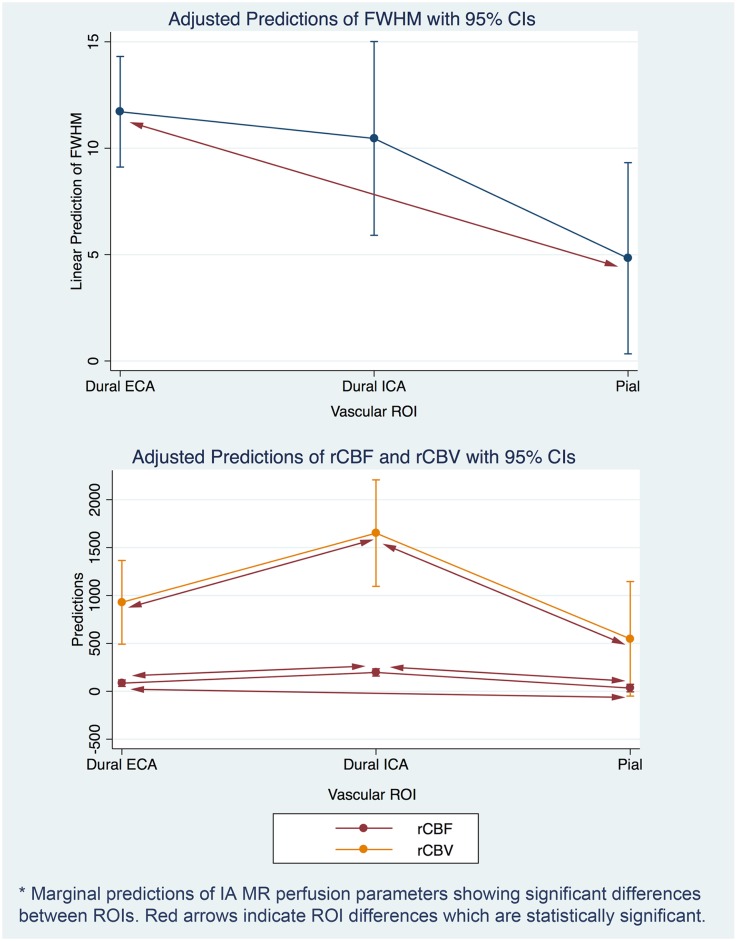
Marginal predictions for IA perfusion parameters showing statistically significant differences between ROIs.

Twelve patients met inclusion criteria during the study period. This included eight women and 4 men who ranged in age from 46 to 78 years. 18 regions-of-interest (ROIs) were uniformly drawn on pre-operative CCA perfusion images representing ECA dural, ICA dural, and pial supplies. Patient and meningioma characteristics are presented in Table 1. Of the 12 meningiomas analyzed by DSA, blood supplies were found to be derived from dural ECA branches (n = 5), dural ICA branches (n = 1), dural ECA and ICA branches (n = 3), and mixed dural-pial branches (n = 3). None of the meningiomas were found to have a pure pial supply. ROIs were drawn for the different types of blood supplies. For meningiomas with a single type of blood supply (i.e. ECA dural, ICA dural), 1 ROI was drawn, and for meningiomas with 2 types of blood supply (i.e. ECA and ICA dural, mixed dural-pial branches), 2 ROIs were drawn resulting in a total of 18 ROIs. IA perfusion curve parameters were calculated for each ROI (Fig 2), and median values were calculated for each type of blood supply. Dural ICA and ECA data were compared to pial data (Table 2). For IA imaging, FWHM was significantly lower for pial ROIs compared to ECA ROIs. There was no significant difference in peak height or MTT. rCBV was found to be higher in ICA compared to pial and ECA. rCBF was found to be significantly higher in the ICA ROI compared to both the ECA and pial ROI and higher in ECA compared to pial ROI. Marginal effects for these differences are illustrated in Fig 3.

**Table 1:** Patient and meningioma characteristics.

Patient	Vascular Supply	Sex	Age	Tumor Location	Tumor Size	Tumor Volume
ME-32	ECA and ICA dural	F	59	Skull base	6.6 x 6.2 x 5.9 cm	241 cm^3^
ME-34	ECA and ICA dural	M	78	Skull base	5.2 x 5.6 x 5.3 cm	154 cm^3^
ME-36	ECA and ICA dural	M	60	Skull base	5.6 x 4.5 x 4.2 cm	106 cm^3^
ME-18	ECA dural	F	58	Skull base	4.3 x 2.7 x 4.4 cm	51 cm^3^
ME-20	ECA dural	M	49	Convexity (falx)	6.2 x 5.3 x 6.6 cm	217 cm^3^
ME-21	ECA dural	F	56	Convexity	4.6 x 5.0 x 5.0 cm	115 cm^3^
ME-25	ECA dural	F	68	Skull base	5.6 x 3.3 x 4.6 cm	85 cm^3^
ME-30	ECA dural	F	67	Skull base	3.2 x 2.5 x 3.5 cm	28 cm^3^
ME-33	ICA dural	F	61	Skull base	6.0 x 6.2 x 6.4 cm	238 cm^3^
ME-03	Mixed dural-pial	M	67	Skull base	4.6 x 3.9 x 4.4 cm	79 cm^3^
ME-28	Mixed dural pial	F	60	Convexity (falx)	2.6 x 2.1 x 2.4 cm	13 cm^3^
ME-40	Mixed dural-pial	F	46	Convexity	7.7 x 5.9 x 6.3 cm	287 cm^3^

**Table 2:** IA MR perfusion curve parameters for dural and pial ROIs.

IA MR perfusion	Mixed-effects REML regression				Dural ECA vs. Dural ICA		Dural ECA vs. Pial		Dural ICA vs. Pial	
	Model Fit		Random Effects							
Perfusion Curve Parameter	Wald Chi2	P-value	Chibar2	P-value	Coefficients [95% CI]	Sig.	Coefficients [95% CI]	Sig.	Coefficients [95% CI]	Sig.
Peak Height	2.71	0.258	0.43	0.256	0.017 (-.143-.178)	0.833	-0.143 (-0.320–0.034)	0.114	0.160 (-0.060–0.380)	0.153
FWHM (s)	5.18	0.075	0.02	0.44	-1.020 (-6.739–4.699)	0.727	-6.538 (-12.169- -.907)	**0.023**	-5.517 (-12.763–1.729)	0.136
rCBV	11.76	**0.003**	2.16	0.071	722.5531 (250.721–1194.385)	**0.003**	-381.066 (-870.123–107.991)	0.127	-1103.619 (-1768.345- -438.893)	**0.001**
rCBF	106.63	**<0.001**	5.70	**0.009**	111.844 (88.396–135.292)	**<0.001**	-51.400 (-75.184- -27.616)	**<0.001**	-163.244 (-196.376- -130.112)	**<0.001**
MTT (s)	1.91	0.384	0.00	1.000	-1.685 (-6.214–2.844)	0.466	-3.403 (-8.442–1.636)	0.186	-1.718(-7.565–4.128)	0.565
rMTT	3.19	0.203	0.97	0.162	-3.198 (-6.721-.325)	0.075	-.572 (-4.298–3.153)	0.763	2.626 (-2.310–7.561)	0.297

**Fig 4:**
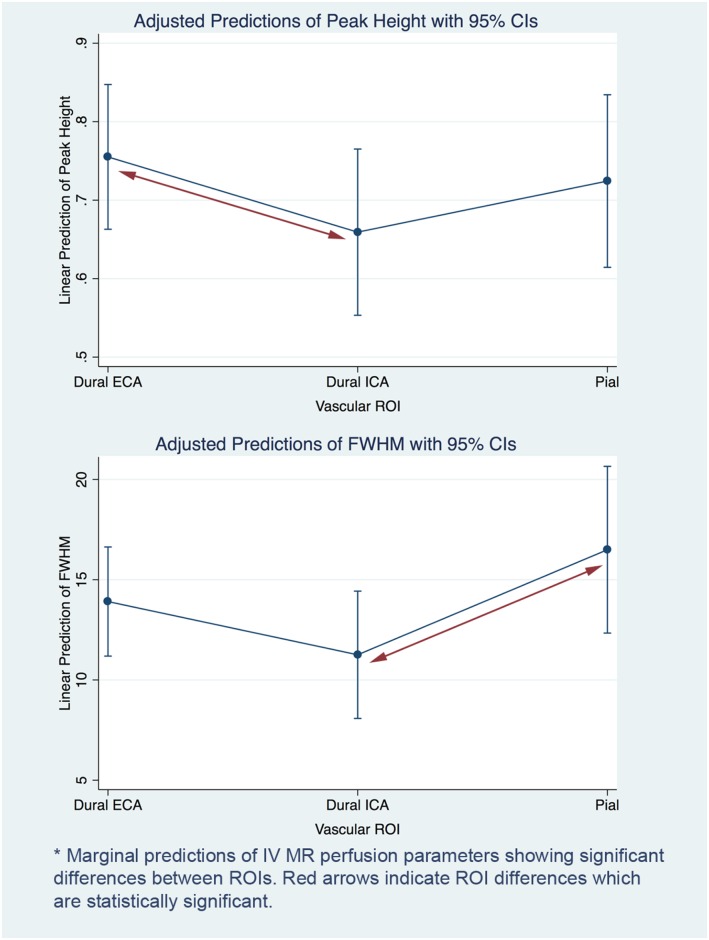
IV imaging perfusion parameter differences between vascular ROIs.

There were two significant differences between dural ECA, ICA and pial data for IV perfusion curve parameters (Table 3). Dural ECA was found to be significantly higher than ICA for Peak Height and FWHM was found to be significantly higher in Pial ROI compared to ICA. Marginal effects for IV parameters are illustrated in Fig 4.

**Table 3:** IV MR perfusion curve parameters for dural and pial ROIs.

IV MR perfusion	Mixed-effects REML regression				Dural ECA vs. Dural ICA		Dural ECA vs. Pial		Dural ICA vs. Pial	
	Model Fit		Random Effects							
Perfusion Curve Parameter	Wald Chi2	P-value	Chibar2	P-value	Coefficients [95% CI]	Sig.	Coefficients [95% CI]	Sig.	Coefficients [95% CI]	Sig.
Peak Height	9.28	**0.01**	10.23	**0.006**	-0.079 (-0.130- -0.027)	**<0.001**	-0.021 (-0.073–0.032)	0.443	0.058 (-0.014–0.130)	0.12
FWHM (s)	4.51	0.1	4.35	0.114	-2.655 (-6.208–0.899)	0.14	2.586 (-0.948–6.120)	0.152	5.240 (0.406–10.074)	**0.03**
rCBV	2.22	0.33	1.77	0.092	-506.740 (-1216.018–202.541)	0.16	157.352 (-596.176–910.880)	0.682	664.091 (-333.211–1661.392)	0.19
rCBF	2.16	0.34	0.67	0.207	-45.753 (-136.169–44.662)	0.32	-62.311 (-161.932–37.310)	0.220	-16.557 (-140.354–107.239)	0.79
MTT (s)	0.06	0.97	47.73	**<0.001**	-.0116 (-0.103–0.080)	0.80	-0.002 (-0.119–0.115)	0.974	0.010 (-0.123–0.142)	0.89
rMTT	0	1.00	103.83	**<0.001**	-.755 (-1.981–0.471)	0.23	0.625 (-0.666–1.917)	0.342	1.381 (-0.393–3.154)	0.13

*4 ROIs were omitted due to <50% signal recovery

**Fig 5:**
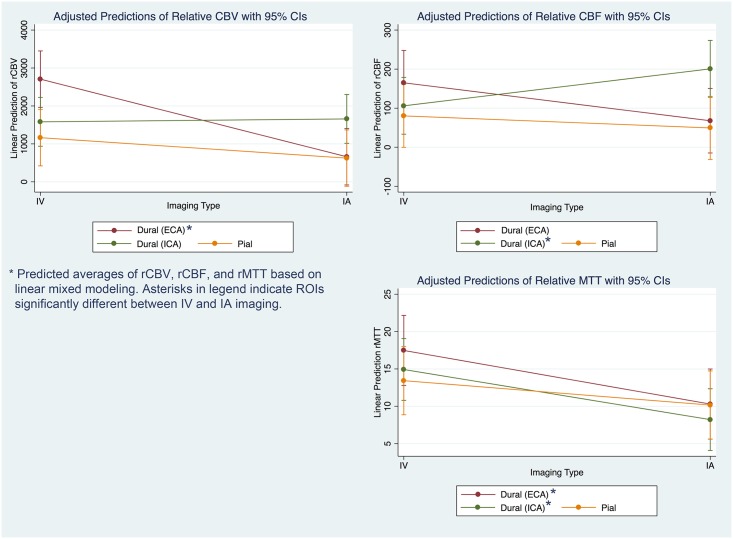
Significant differences in perfusion parameters between IA and IV imaging.

Perfusion parameter differences between IV and IA for each ROI are presented in Table 4. rCBV was found to be significantly higher in IV MR imaging compared to IA in ECA. rCBF was found to be significantly higher in IA compared to IV in ICA, and ECA and ICA were both significantly higher in IV compared to IA imaging for rMTT. Marginal effects of these comparisons are presented in Fig 5.

**Table 4:** IA vs. IV MR perfusion curve parameters by ROI.

IA vs. IV MR perfusion	Mixed-effects REML regression				Dural ECA		Dural ICA		Pial	
	Model Fit		Random Effects							
Perfusion Curve Parameter	Wald Chi2	P-value	Chibar2	P-value	Coefficients [95% CI]	Sig.	Coefficients [95% CI]	Sig.	Coefficients [95% CI]	Sig.
Peak Height	8.6	0.28	0.33	0.284	0.029 (-.256-.315)	0.84	.168 (-.091-.426)	0.203	-.182 (-.468-.103)	0.21
FWHM (s)	16.41	**0.02**	0.42	0.257	-8.382 (-19.416–2.650)	0.14	-.163 (-7.82–7.495)	0.967	-8.819 (-17.465- -.173)	0.05
rCBV	24.39	**<0.001**	0.00	1.000	-1810.117 (-3054.247- -565.987)	**<0.001**	313.467 (-814.278–1441.212)	0.586	-302.547 (-1546.677–941.584)	0.63
rCBF	17.28	**0.02**	2.30	0.065	-29.047 (-148.396–90.302)	0.63	162.919 (54.672–271.166)	**0.003**	37.220 (-82.129–156.569)	0.54
MTT (s)	7.36	0.39	0.10	0.3775	-5.064 (-11.069–0.945)	0.10	-3.324 (-8.768–2.120)	0.231	-3.721 (-9.726–2.285)	0.23
rMTT	16.41	**0.02**	2.36	0.0621	-7.89 (-14.601- -1.170)	**0.02**	-7.422 (-13.513- -1.331)	**0.017**	-3.980 (-10.695–2.736)	0.25

## Discussion

MRI offers advantages over DSA including the ability to characterize tissue physiology with diffusion and perfusion. MRI can provide quantitative data in contrast to the qualitative nature of DSA, which can be used to identify targets that would benefit from embolization. Martin et al. have previously demonstrated feasibility of IA MR perfusion of meningiomas in the perioperative setting[14]. Currently, this is relevant in the context of reducing intraoperative bleeding; however, embolization could potentially be effective as stand alone therapy in selected patients[15], particularly with the development of new therapeutic agents.

Perfusion curve parameters that would be useful to characterize meningioma vasculature should be easy to calculate and reproducible. FWHM, previously described in stroke imaging, is a useful measure to characterize the permeability of tumor blood supply. Peak height is thought to be a measure of tumor vascularity as it correlates well with cerebral blood volume[10, 16]. MTT quantifies the time contrast spends in the capillaries, while rCBV quantifies blood within a mass of tissue. Dividing the latter by the former yields rCBF.

For IA MR perfusion data, differences in perfusion curve parameters were found for, rCBV and rCBF between ICA and ECA dural supply, suggesting these IA MR perfusion parameters may be sensitive enough to distinguish between these different types of dural supplies. ICA dural variants, such as an anterior falx artery arising from the ophthalmic artery (Fig 3), are readily apparent on DSA but not amenable to embolization as reflux of embolic agents could compromise blood flow to the retina.

Comparison of ECA dural and pial perfusion curve parameters, however, reveals significantly reduced FWHM for pial ROIs from IA MR perfusion. Additionally, ICA ROIs were found to have significantly higher rCBV and rCBF compared to both ECA and pial ROIs. This reflects known differences in the blood brain barrier, which is largely absent in dural branches but present in pial branches. These differences are also apparent on qualitative examination of the perfusion curves for meningiomas with mixed dural-pial supply. The progressive broadening of the IA perfusion curve as ROIs are moved from pial to dural territory can be explained by blood brain barrier physiology. As predicted, there were no differences in peak height, as this relates to tumor vascularity and not necessarily blood brain barrier permeability.

Characteristics of MR perfusion curves are dictated by the concentration of contrast agent perfusing a given territory over time. With IA injection, the injected bolus remains sharply defined with rapid arrival and rapid clearance. The concentration of this first-pass IA bolus is much higher than that of the second-pass that has been diluted throughout the vascular system. In comparison, the contrast bolus delivered by IV injection is dispersed in the vasculature before it reaches the brain. Furthermore, the relative intensity of the second pass of an IV bolus can be similar to that of the first pass, inhibiting the return of signal intensity to baseline. This is demonstrated by the IV perfusion curves suggesting higher permeability compared to the IA curves. Comparison of IV MR perfusion curve data was limited by poor signal recovery in many ROIs. A significant difference for Peak Height was found when comparing ICA vs. ECA dural curves, and FWHM was found significantly different between ICA vs. pial curves. While IV MR perfusion is more convenient and accessible, these data do not support its ability to triage patients to preoperative embolization as well as IA MR perfusion since IA MR perfusion seems more sensitive to perfusion parameter differences between ROIs. The results of this study suggest a confirmatory role from IA MR perfusion can enhance confidence that a vascular pedicle supplying a pedicle can be safely embolized. These techniques may also allow evaluation of intratumoral recollateralization from non-embolized arteries following particulate embolization of one feeding artery. As such, this technique may be useful in the future context of chemoembolization of meningiomas, wherein cytotoxic drug delivery could be significantly affected by changes in intratumoral blood flow.

A limitation of this study is the small number of patients analyzed in this exploratory evaluation of these techniques. Despite identifying only 3 patients harboring meningiomas with pial supply, we were still able to demonstrate significant differences between pial and dural ROIs. A larger number of patients would be necessary to validate our findings. Additionally, MR signal saturated to the noise floor on IA injections, imposing an artificial limit on peak height and making it difficult to define an arterial input function. This limited us to the utilization of only relative measures for CBV and CBF. The IA data similarly did not permit calibration from remote white matter, which commonly was not within the IA injections distribution territory. The concordance between the IV and IA perfusion measures therefore relies on the consistency of the administered bolus and imaging approach, which is an acknowledged limitation of the study. Finally, while the mixed effects modeling used is designed to capture non-normal outcomes such as peak heights in our perfusion data, this is a limitation of these methods that cannot be overcome in the current analysis. The utilization of a lower Gadolinium concentration in the IA injections would reduce these saturation effects.

## Conclusion

In summary, in contrast to DSA, selective IA MR perfusion can provide quantitative measures to identify pial supply that might preclude meningioma embolization. As such, it could serve as an adjunct to DSA in evaluation of tumor supply by providing physiologic data in current practice and could be an important component of tumor evaluation during completely MRI-guided tumor embolization if interventional MRI supplants x-ray guided embolization in the future[17].
